# A two-stage computational framework for identifying antiviral peptides and their functional types based on contrastive learning and multi-feature fusion strategy

**DOI:** 10.1093/bib/bbae208

**Published:** 2024-05-05

**Authors:** Jiahui Guan, Lantian Yao, Peilin Xie, Chia-Ru Chung, Yixian Huang, Ying-Chih Chiang, Tzong-Yi Lee

**Affiliations:** School of Medicine, The Chinese University of Hong Kong, Shenzhen, 2001 Longxiang Road, 518172 Shenzhen, China; Kobilka Institute of Innovative Drug Discovery, School of Medicine, The Chinese University of Hong Kong, 2001 Longxiang Road, 518172 Shenzhen, China; School of Medicine, The Chinese University of Hong Kong, Shenzhen, 2001 Longxiang Road, 518172 Shenzhen, China; School of Science and Engineering, The Chinese University of Hong Kong, 2001 Longxiang Road, 518172 Shenzhen, China; Kobilka Institute of Innovative Drug Discovery, School of Medicine, The Chinese University of Hong Kong, 2001 Longxiang Road, 518172 Shenzhen, China; Department of Computer Science and Information Engineering, National Central University, 320317 Taoyuan, Taiwan; School of Medicine, The Chinese University of Hong Kong, Shenzhen, 2001 Longxiang Road, 518172 Shenzhen, China; School of Medicine, The Chinese University of Hong Kong, Shenzhen, 2001 Longxiang Road, 518172 Shenzhen, China; Kobilka Institute of Innovative Drug Discovery, School of Medicine, The Chinese University of Hong Kong, 2001 Longxiang Road, 518172 Shenzhen, China; Institute of Bioinformatics and Systems Biology, National Yang Ming Chiao Tung University, 300093 Hsinchu, Taiwan; Center for Intelligent Drug Systems and Smart Bio-devices (IDS2B), National Yang Ming Chiao Tung University, 300093 Hsinchu, Taiwan

**Keywords:** antiviral peptides, drug discovery, contrastive learning, multi-feature fusion strategy, sequence analysis

## Abstract

Antiviral peptides (AVPs) have shown potential in inhibiting viral attachment, preventing viral fusion with host cells and disrupting viral replication due to their unique action mechanisms. They have now become a broad-spectrum, promising antiviral therapy. However, identifying effective AVPs is traditionally slow and costly. This study proposed a new two-stage computational framework for AVP identification. The first stage identifies AVPs from a wide range of peptides, and the second stage recognizes AVPs targeting specific families or viruses. This method integrates contrastive learning and multi-feature fusion strategy, focusing on sequence information and peptide characteristics, significantly enhancing predictive ability and interpretability. The evaluation results of the model show excellent performance, with accuracy of 0.9240 and Matthews correlation coefficient (MCC) score of 0.8482 on the non-AVP independent dataset, and accuracy of 0.9934 and MCC score of 0.9869 on the non-AMP independent dataset. Furthermore, our model can predict antiviral activities of AVPs against six key viral families (Coronaviridae, Retroviridae, Herpesviridae, Paramyxoviridae, Orthomyxoviridae, Flaviviridae) and eight viruses (FIV, HCV, HIV, HPIV3, HSV1, INFVA, RSV, SARS-CoV). Finally, to facilitate user accessibility, we built a user-friendly web interface deployed at https://awi.cuhk.edu.cn/∼dbAMP/AVP/.

## INTRODUCTION

Viral diseases have long posed a significant challenge to global public health, profoundly impacting human health and socio-economic structures [1]. Particularly in recent years, the emergence and spread of novel viruses like severe acute respiratory syndrome coronavirus (SARS-CoV-2) have highlighted the challenges posed by new, more threatening virus strains to global health security [2, 3]. Although existing vaccines and antiviral drugs have made progress in controlling certain viral diseases, the rapid mutation of viruses, their minuscule structure and complex mechanisms pose challenges [4]. These include the complexity of antiviral treatments, the emergence of viral resistance and the lack of effective treatments for some stubborn viruses [5]. Moreover, the high cost and lengthy process of developing new vaccines compound the difficulty in rapidly responding to emerging viral diseases[6].

In this backdrop, antiviral peptides (AVPs) have gained widespread attention as an emerging therapeutic strategy due to their unique antiviral mechanism, low risk of resistance and potentially broad antiviral spectrum [7, 8]. These AVPs, whether naturally derived or synthetically produced, have demonstrated potential in inhibiting viral attachment, blocking virus-host cell fusion, and interfering with viral replication[9]. The mechanisms of action of AVPs are diverse and complex. For instance, Mucroporin-M1 can disrupt the viral envelope, reducing the virus’s ability to infect host cells [10]. Some AVPs such as HR2-M2, EK1, EK1C4 and TMPRSS2 primarily inhibit the spike protein of coronaviruses, hindering protein-mediated fusion and activation of the S protein [11]. In addition, AVPs like Human Defensin-5 (HD5) protect ACE2 receptors on host cells, while P9 inhibits the acidification of late endosomes, crucial for preventing the release of viral RNA. Furthermore, AVPs such as RTD-1 can activate the host’s immune response, enhancing defenses against viruses [12]. These varied and effective mechanisms highlight the potential of AVPs as a powerful tool in antiviral therapy. Building upon these insights, the advancement of the pseudo-isolated $\alpha $-helices (mPIH) platform alongside antimicrobial peptides (AMPs) marks a substantial progression in peptide-based therapeutic strategies [13, 14]. The mPIH system can leverage stabilized $\alpha $-helical structures to enhance the specificity and stability of AVPs, combining the benefits of small peptides with the structural integrity of protein-based therapeutics. Simultaneously, insights gained from AMP research, especially their function as a natural defense mechanism and their cutting-edge nanotechnology-based delivery methods [15], have been instrumental in refining AVP strategies. This integrated approach enhances the effectiveness and application of AVPs, offering a promising avenue for antiviral therapies.

However, identifying peptides through traditional laboratory methods is a time-consuming and expensive task [16]. Addressing this challenge, machine learning (ML) algorithms have been introduced as a prospective strategy to accelerate peptide research and applications because of their efficient characterization capabilities [17, 18]. In recent years, ML has made significant progress in the prediction and identification of AVPs. Initially, Thakur et al. [19]. developed an AVPpred model utilizing support vector machine (SVM) and physicochemical properties of the AAindex database, which demonstrated good prediction accuracy on a benchmark dataset. After that, Chang et al. [20]. further developed a model with better performance using amino acid composition and aggregation propensity features via Random Forest (RF) algorithm. Then, Lissabet et al. [21]. introduced AntiVPP 1.0, based on RF and diverse physicochemical properties. Based on these single learning methods, Schaduangrat et al. [22]. developed the integrated classification method Meta-iAVP, which combines the predicted outputs of six different hypothesis learning methods to form an ensemble classifier. Furthermore, Li et al. [23]. employed a two-channel deep neural network, which combined a long- and short-term memory network and a convolutional neural network developed DeepAVP for identifying AVPs. Afterwards, Akbar et al. [24]. combined discrete wavelet transform and k-segmentation techniques and utilized shapley additive explanations to pick the optimal features, which were then validated by five ML classifiers. A final ensemble learner was constructed by inputting the predicted labels into a genetic algorithm.

While these ML-based tools have achieved certain breakthroughs in the identification of AVPs, they still possess limitations. First, the lack of comprehensiveness and completeness of the datasets utilized in previously developed models limits the broad applicability of these models. Second, most of the existing methods mainly use a limited set of feature descriptors, often ignoring the key evolutionary information and physicochemical properties of peptides, which affects the accuracy and generalizability of the models. Finally, most of the existing methods are still based on traditional ML algorithms, whereas the use of deep learning algorithms can provide a deeper understanding of peptide properties.

**Figure 1:**
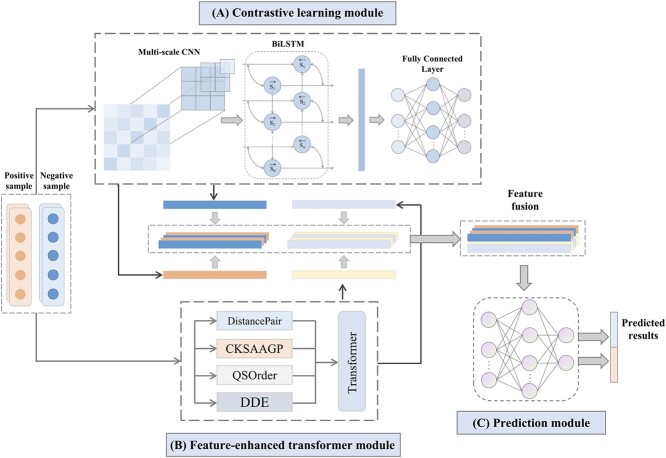
Framework of the proposed model. The model consists of three main modules: (**A**) Contrastive learning module. (**B**) Feature-enhanced transformer module. (**C**) Prediction module.

In this study, we proposed an innovative two-stage method for the identification of AVPs. The first stage is designed to identify AVPs in complex samples, while the second stage focuses on recognizing AVPs targeting specific viral families and viruses. This predictor was developed based on contrastive learning and a multi-feature fusion strategy. Specifically, we constructed a contrastive learning module based on sequence information and a feature enhancement module based on peptide characteristics. By fusing these multifaceted features, we developed the final prediction model. The framework of the proposed model is shown in Figure 1, which can be divided into three modules: (i) contrastive learning module; (ii) feature-enhanced transformer module and (iii) Prediction module. In the first module, our model utilizes binary, BLOSUM62 and Zscale encoding to transform the original sequence into a numeric matrix. Then, the obtained matrix is fed into a network consisting of multi-scale convolutional neural network (CNN) and Bidirectional Long Short-Term Memory (BiLSTM) . Subsequently, the extracted features are fed into the optimization module to maximize the consistency (inconsistency) between similar (dissimilar) AVPs using contrastive Learning. Finally, the enhanced features extracted by the second module are fused with the contrastive learning features and then the fused features are fed into the prediction module to identify whether a given sequence is an AVP and its functional type.

Our study contributes to the early identification of AVPs by offering a more effective recognition method, thereby reducing labor costs. Compared to existing methods, our model demonstrates significant improvements in predictive performance. More importantly, by emphasizing key features and sequence positions, we have enhanced the interpretability of the model, which is an significant contribution to the insight into the critical characteristics of AVPs. Additionally, our study provides valuable insights for the design and development of future antiviral drugs. With the continuous advancement of ML predictive tools, our model is expected to substantially contribute to the development of innovative therapeutic strategies against various viruses.

## MATERIALS AND METHODS

### Dataset preparation

This study established two datasets: non-AVP and non-AMP, with both utilizing AVPs as positive samples. AVPs were extracted from multiple databases including dbAMP[25], AVPdb[7], DRAMP[26], DBAASP[27] and HIPdb[28], retaining sequences with a length between 8 and 130, while sequences containing invalid amino acids were excluded. The preprocessed AVPs comprises 2662 unique sequence records targeting various viruses. Negative samples for the non-AVP dataset were collected from the dbAMP, DBAASP and DRAMP databases, which do not include virus-specific records. Negative samples for the non-AMP dataset were retrieved from UniProt[29], filtering sequences with the attribute keywords: ’toxic,’ ’membrane,’ ’secretory,’ ’defensive,’ ’antibiotic,’ ’anticancer,’ ’antiviral,’ ’antifungal.’ These negative sample sequences were de-duplicated using the Cluster Database at High Identity with Tolerance (CD-HIT) tool with a threshold set at 40%, eventually selecting the same number of sequences as the positive samples to construct balanced datasets, totaling 2,662.

Among AVPs, most sequences can target certain viruses, as well as families of viruses. Based on the multifunctionality of AVPs, we divided the data into six viral families: Coronaviridae, Retroviridae, Herpesviridae, Paramyxoviridae, Orthomyxoviridae and Flaviviridae, and into eight targeted viruses: Feline Immunodeficiency Virus (FIV), Human Immunodeficiency Virus (HIV), Hepatitis C Virus (HCV), Human Parainfluenza Virus Type 3 (HPIV3), Herpes Simplex Virus Type 1 (HSV1), Influenza A Virus (INFVA), Respiratory Syncytial Virus (RSV) and SARS-CoV. These functional types were selected under the criteria of having at least 100 sequence records for viral families and at least 80 sequence records for targeted viruses, ensuring the validity of our training set and the feasibility of model training. Based on the viral families and targeted viruses, we established six and eight multifunctional classified datasets, with specific details listed in Tables 1 and 2.

**Table 1:** Overview of multifunctional classified datasets – viral Families

Viral family	Coronaviridae	Retroviridae	Herpesviridae	Paramyxoviridae	Orthomyxoviridae	Flaviviridae
Positive samples	184	995	267	272	113	489
Negative samples	2478	1667	2395	2390	2549	2173

**Table 2:** Overview of multifunctional classified datasets – targeted Viruses

Targeted virus	FIV	HIV	HCV	HPIV3	HSV1	INFVA	RSV	SARS-CoV
Positive samples	101	867	438	87	213	112	119	137
Negative samples	2561	1795	2224	2575	2449	2550	2543	2525

In addition, we also utilized two datasets from previous research: set 1 contains 604 experimentally validated AVPs and 452 validated non-AVPs; set 2 includes 604 validated AVPs and 604 non-validated non-AVPs. After removing sequences with invalid amino acids, the specific numbers of samples in sets 1 and 2 are summarized in Table 3.

**Table 3:** Summary of sets 1 and 2 datasets

Datasets	Train/test sets	Positive samples	Negative samples
Set 1	Train	540	400
	Test	60	44
Set 2	Train	540	541
	Test	60	58

To convert peptide sequences into a numerical format suitable for model input, we employed a series of sequence encoding methods, including Binary, BLOSUM62, Zscale, DistancePair, CKSAAGP, QSOrder and DDE, for feature preparation. These encoding methods allow for a multi-perspective representation of peptides.

### Framework of the Proposed Model Contrastive learning module

#### Sequence encodings

In the contrastive learning module, we employed three sequence encoding methods: Binary, BLOSUM62 and Zscale.

The Binary encoding represents each amino acid as a unique 20-dimensional binary vector, effectively distinguishing it from the other 19 standard amino acids.

The BLOSUM62 encoding converts the peptide sequence into a numeric vector reflecting the rate of amino acid substitution. Each amino acid is converted into a 20-dimensional vector based on the likelihood of its substitution[30].

The Zscale encoding quantifies the physical and chemical properties of amino acids in protein sequences. It is based on the Z-scale normalization method used to normalize and compare the various biophysical and chemical properties of amino acids. Each amino acid is represented as a 5-dimensional vector[31].

By concatenating these three encodings, we get a L*45 feature matrix, where L represents the length of the sequence. The details of peptide encodings can be found in the supplementary materials.

#### Model architecture

In the contrastive learning module, we utilized a multi-scale CNN + BiLSTM architecture as its superior capability in processing sequential data. The multi-scale CNN captures multi-scale features through convolutional kernels of varying sizes, while the BiLSTM effectively captures long-term dependencies in the sequences.

To illustrate the advantages of our selected network architecture, we compared it with three other networks: traditional CNNs [32], long short-term memory (LSTM) networks [33] and gated recurrent unit (GRU) networks [34], for a more thorough comparison. Table 4 summarizes the characteristics of each network. The multi-scale CNN + BiLSTM offers the best balance for handling AVP sequences, effectively capturing complex sequence features while maintaining a relatively reasonable computational complexity. Although the GRU provides a lower computational demand alternative capable of effectively handling long-term dependencies, the multi-scale CNN + BiLSTM performs better with extremely complex sequence features.

**Table 4:** Comparison of different network architectures

Network architecture	Advantages	Disadvantages
Traditional CNNs	Fewer parameters,	Struggles to capture
	easier to train	long-term dependencies
LSTM	Excellent at capturing	Difficult to manage with large datasets,
	long-term dependencies	longer training times
GRU	Good long-term dependency handling with	Less effective at managing extremely complex
	lower computational demands than LSTM	sequence features compared to Multi-scale CNN+BiLSTM
Multi-scale	Captures multi-scale features	Relatively complex,
CNN + BiLSTM	and long-term dependencies	requires more computational resources

Initially, we used eight differently shaped convolutional kernels to process the encoded sequences[35]. These kernels are designed to match the encoding dimensions of amino acids. As the number of convolution layers increases, the size of the filters also progressively enlarges. Each layer employs 64 kernels of the same shape to extract convolutional features. For an input sample X, the mathematical representation of the convolution layer is: 


(1) \begin{align*} y_{i}^{k} = \text{Relu}\left(\sum_{p=0}^{P-1} \sum_{q=0}^{Q-1} w_{pq}^{k} \cdot x_{i+p, q} + b^{k}\right)\end{align*}

where, $\text{Relu}(x) = \max (0, x)$. $w^{k}$ is the weight matrix of the kth convolutional kernel, with dimensions $P \times Q$.

After the convolution, the dimensionality of the feature maps increases. To obtain a compact feature representation, we employ max-pooling. This pooling strategy selects the most significant features from the output of the last convolution layer, mathematically described as: 


(2) \begin{align*} \text{pooling}(X)_{i,k} = \max(X_{iM,k}, X_{iM+1,k}, \ldots, X_{iM+M-1,k})\end{align*}

where, $X$ represents the input feature matrix, $i$ is the row index in the feature matrix, $k$ is the column index corresponding to different feature channels and $M$ is the window size of the pooling operation.

Features obtained after pooling are reshaped to fit the input format of the BiLSTM [36]. The LSTM is a special type of recurrent neural network, adept at handling long sequence data by capturing dependencies across intervals and delays. The BiLSTM captures dependencies both forward and backward in the input sequence, providing a more comprehensive view of sequence information. The core formulas for the LSTM are: 


(3) \begin{align*} f_{t} = \sigma(W_{f} \cdot [h_{t-1}, x_{t}] + b_{f}) \end{align*}

(4) \begin{align*} i_{t} = \sigma(W_{i} \cdot [h_{t-1}, x_{t}] + b_{i}) \end{align*}

(5) \begin{align*} \tilde{C}_{t} = \tanh(W_{C} \cdot [h_{t-1}, x_{t}] + b_{C}) \end{align*}

(6) \begin{align*} C_{t} = f_{t} \cdot C_{t-1} + i_{t} \cdot \tilde{C}_{t} \end{align*}

(7) \begin{align*} o_{t} = \sigma(W_{o} \cdot [h_{t-1}, x_{t}] + b_{o}) \end{align*}

(8) \begin{align*} h_{t} = o_{t} \cdot \tanh(C_{t}) \end{align*}

where $f_{t}$, $i_{t}$ and $o_{t}$ correspond to the activation vectors for the forget gate, input gate and output gate at time $t$, managing the sequential information flow. $\tilde{C}_{t}$ and $C_{t}$ are candidate and actual cell states at time $t$, representing the memory of the network. $h_{t}$ is the hidden state vector summarizing the processed information up to time $t$. The weight matrices $W_{f}, W_{i}, W_{C}, W_{o}$, and bias vectors $b_{f}, b_{i}, b_{C}, b_{o}$ are fundamental components that drive the gate operations and memory updates within the LSTM, facilitated by the sigmoid function $\sigma $ and the hyperbolic tangent function $\tanh $, which modulate the activations and updates of the cell states.

In BiLSTM setup, we can get the output results from both forward and backward directions,denoted as $h_{t}^{forward}$ and $h_{t}^{backward}$, respectively. Finally, forward and backward hidden states are concatenate.

#### Contrastive loss function

The objective of contrastive learning is to learn a feature space where samples with similarities are brought closer, while those with dissimilarities are distanced apart [37]. To achieve this learning process, a contrastive loss function is employed during model training. In this context, consider two samples represented as $ x_{1} $ and $ x_{2} $ within the dataset. Their relationship is defined by a binary label $ y $: $ y = 0 $ indicates similarity between the samples, whereas $ y = 1 $ denotes dissimilarity. The spatial distance in the feature space is quantified by the Euclidean distance metric with the following formula: 


(9) \begin{align*} D_{w}(x_{1}, x_{2}) = \lVert x_{1} - x_{2} \rVert_{2}\end{align*}

The contrastive loss function comprises two main components:

For similar samples (when $ y = 0 $), the loss, termed as Loss for Positive Pairs, seeks to minimize their distance in the feature space. Mathematically, this is represented as: 


(10) \begin{align*} L_{positive} = (1 - y) \times D_{w}(x_{1}, x_{2})^{2}\end{align*}

On the contrary, for dissimilar samples (when $ y = 1 $), the loss, or the Loss for negative pairs, aims to ensure they are at least a preset distance apart, defined by a ’margin.’ This is formulated as: 


(11) \begin{align*} L_{negative} = y \times \max(0, margin - D_{w}(x_{1}, x_{2}))^{2}\end{align*}

Aggregating these components yields the overall Contrastive Loss: 


(12) \begin{align*} L_{contrastive} = L_{positive} + L_{negative}\end{align*}

Further, this loss is averaged across all sample pairs in a given batch: 


(13) \begin{align*} L = \frac{1}{N} \sum_{i=1}^{N} L_{contrastive}\end{align*}

### Feature-enhanced transformer module

#### Sequence encodings

In the feature-enhanced transformer module, we utilized four sequence encoding methods: DistancePair, CKSAAGP, QSOrder and DDE.

The PseAAC of distance-pair and reduced alphabet (DistancePair) encoding describes the pairwise distances between amino acids in a protein sequence, reflecting their relative positions and potential interactions, and is closely related to the structure of proteins [38].

The composition of k-spaced amino acid group pairs (CKSAAGP) encoding divides a protein sequence into overlapping groups of amino acids and calculates their frequencies to capture localized patterns and identify functional regions or active sites [39].

The quasi-sequence order (QSOrder) encoding employs a quasi-sequence descriptor approach. It analyzes the overall composition of amino acids and integrates information about their position in the protein sequence, fusing local and global features [40, 41].

The dipeptide deviation from expected mean (DDE) encoding quantifies the deviation between the observed frequency of a dipeptide and its theoretically anticipated occurrence based on codon frequencies. A significant divergence from expected frequencies for a specific dipeptide may hint at underlying evolutionary, functional, or structural rationales [42].

By combining these encoding methods, each sequence is converted into a 566-dimensional feature vector. The details of peptide encodings can be found in the Supplementary materials.

#### Transformer

The Transformer architecture excels in diverse tasks, especially in capturing complex feature interactions. At its core is the self-attention mechanism, which enables the model to capture dependencies between input features [43]. Considering the biological attributes of protein data, the feature space is often high-dimensional and the relationships between these features can be crucial [44]. The Transformer, with its self-attention mechanism, can effectively handle these high-dimensional feature vectors and discern the interrelations among them. The formula is as follows: 


(14) \begin{align*} \text{Attention}(Q, K, V) = \text{softmax} \left( \frac{QK^{T}}{\sqrt{d_{k}}} \right) V\end{align*}

Where $ Q $, $ K $ and $ V $ represent the query, key, and value matrices, respectively, and $ d_{k} $ denotes the dimension of the key.

In addition, A significant attribute of the Transformer model is its interpretability, which is primarily attributed to its attention-based mechanism. By analyzing the attention weights, we can gain a deep understanding of the features the model deems important during the decision-making process.

### Imbalanced learning strategy

In the second phase of our model’s prediction task, our objective is to classify based on the multifunctionality of AVPs. However, as illustrated in Table 1 for the viral family datasets and Table 2 for the targeted virus datasets, there is a pronounced imbalance in the distribution of positive and negative samples. This imbalance can introduce a bias in the model’s predictions, skewing them towards the overrepresented categories. To address this problem, we incorporated imbalance learning strategies to ensure a fairer representation of all categories during prediction.

In this study, we employed the focal loss function. Compared to the traditional cross-entropy loss, focal Loss is effective in dealing with unbalanced categorization scenarios [45]. Its primary function is to reduce the weight of easily categorized samples and heightens the model’s focus on samples that are challenging to categorize. This approach not only improves the overall performance of the model, but also ensures reliable predictions for all categories[46]. The formulation of focal loss is: 


(15) \begin{align*} L_{Focal} = -\alpha_{t} (1 - p_{t})^{\gamma} \log(p_{t})\end{align*}

Here, $p_{t}$ denotes the model’s predicted probability for the correct class. Meanwhile, $\alpha _{t}$ is a balancing factor for the positive and negative sample weights, and $\gamma $, referred to as the focusing parameter, is a tunable factor. By fine-tuning these parameters, we can further enhance the model’s performance in the face of imbalanced data.

### Prediction module

In the prediction module, we extracted the final hidden layer output as representative features from the Contrastive learning module (CLM) and the Feature-enhanced transformer (FTM) module, respectively. For each input sample $ x_{i} $, its hidden layer output through CLM is denoted as $ h^{(CLM)}_{i} $, and its hidden layer output through FTM is denoted as $ h^{(FTM)}_{i} $.

By fusing the features learned from different models, we get our final input features. The feature fusion for each sample is given by the following equation: 


(16) \begin{align*} h^{(fused)}_{i} = \text{concat}(h^{(CLM)}_{i}, h^{(FTM)}_{i})\end{align*}

Subsequently, we input these fused features into a fully connected network (FCN). The output prediction of this classifier can be expressed as: 


(17) \begin{align*} y_{pred} = \text{FCN}(h^{(fused)})\end{align*}

### Model training and experimental setup

To ensure reliable predictive performance, our model was trained for 150 epochs with an initial learning rate of 0.001. We implemented a learning rate scheduler to reduce the rate by a factor of 0.95 every five epochs, promoting optimal convergence and fitting. Additionally, we utilized a 5-fold cross-validation approach for optimal parameter selection, enhancing reliability and generalizability. The Adam optimizer was employed for efficient fitting, complemented by an early stopping strategy to mitigate overfitting by ceasing training when no accuracy improvements were observed after a specified number of epochs. To further prevent overfitting, dropout was incorporated, randomly excluding neurons during training to avoid dependency on particular features.

Our experimental development was based on Python[47], and we used the PyTorch framework to construct and train the model[48]. In addition, we employed the iFeatureOmega package to obtain some sequence encodings, which include DistancePair, CKSAAGP and QSOrder[49]. Lastly, the training process was conducted using four Nvidia 2080 Ti GPUs.

### Evaluation metrics

In this study, we utilized Accuracy, Sensitivity, Specificity, Matthew’s correlation coefficient (MCC) [50], and geometric mean of sensitivity and specificity (G-mean) to evaluate the performance of the presented method. They are defined as follows: 


(18) \begin{align*} Accuracy &= \frac{TP+TN}{TP+TN+FP+FN} \end{align*}

(19) \begin{align*} Sensitivity &= \frac{TP}{TP+FN} \end{align*}

(20) \begin{align*} Specificity &= \frac{TN}{TN+FP} \end{align*}

(21) \begin{align*} MCC &= {\frac{TP\times TN-FP\times FN}{\sqrt{(TP+FP)(TP+FN)(TN+FP)(TN+FN)}}} \end{align*}

(22) \begin{align*} G\text{-}mean &= \sqrt{Sensitivity \times Specificity} \end{align*}

where TP, TN, FP and FN denote true positives, true negatives, false positives and false negatives, respectively.

## RESULTS AND DISCUSSION

### Overview of amino acid distributions

To explore the differences between the various samples, we analyzed their amino acid composition. Considering the physicochemical properties of the amino acids, we classified them into five different groups. As shown in Figure S1(A), on the non-AVP dataset, AVPs are rich with acidic amino acids Aspartic (D) and Glutamic (E). In contrast, non-AVPs show a higher content of basic amino acids Arginine Lysine (K), along with Glycine (G). Meanwhile, the results from the non-AMP dataset, as shown in Figure S1(B), indicate a higher proportion of the hydrophobic amino acid Leucine (L) and Tryptophan (W) in AVPs. Notably, compared to the non-AVP dataset, the non-AMPs contained a higher proportion of acidic amino acids Aspartic (D) and Glutamic (E).

In addition, we compared the amino acid compositions of AVPs against different viral families and targeted viruses. As shown in Figure S2(A), AVPs targeting the Retroviridae family appear to have higher abundance in glycine (G) and glutamic acid (E). The Orthomyxoviridae family shows a higher content in leucine (L), while the Flaviviridae family exhibits a notably high valine (V) and lysine (K) content compared to other families. The Paramyxoviridae family stands out with a significant abundance in serine (S). The Herpesviridae family contains high levels of arginine (R). The Coronaviridae family does not exhibit any specific amino acid in high abundance. As illustrated in Figure S2 (B), AVPs targeting the FIV virus have a higher abundance of tryptophan (W). HCV shows significantly higher contents of valine (V) and lysine (K) compared to other viruses. INFVA is notably richer in proline (P) than other viruses. HPIV3 and RSV have a significantly higher serine (S) content, and it is noteworthy that HPIV3 lacks cysteine (C), methionine (M), and phenylalanine (F). HIV, HSV1 and SARSCoV do not exhibit a significantly higher abundance of any amino acids. We also observed that AVPs have very low contents of cysteine (C), methionine (M), phenylalanine (F), tryptophan (W) and histidine (H).

### Performance evaluation

#### Model performance of Stages 1 and 2

In the first stage of the study, the performance of our model on non-AVP and non-AMP datasets is shown in Table 5. The model demonstrated excellent performance on both datasets. Specifically, on the non-AVP dataset, our model achieved accuracy of 0.9240, sensitivity of 0.9343, specificity of 0.9137 and MCC score of 0.8482. On the non-AMP dataset, our model also performs outstandingly with accuracy of 0.9934, sensitivity of 0.9944, specificity of 0.9925 and MCC score of 0.9869. These results indicate that the model is effectively distinguishing between AVPs and non-AVPs under different negative sample conditions, showcasing the model’s generalization ability and strong predictive performance.

**Table 5:** Performance summary on independent dataset of non-AVP and non-AMP datasets

Dataset	Accuracy	Sensitivity	Specificity	MCC
non-AVP	0.9240	0.9343	0.9137	0.8482
non-AMP	0.9934	0.9944	0.9925	0.9869

During the second stage, our model can classify the functional types of AVP into six viral families: Coronaviridae, Retroviridae, Herpesviridae, Paramyxoviridae, Orthomyxoviridae and Flaviviridae, as well as eight targeted viruses: Feline Immunodeficiency Virus (FIV), Human Immunodeficiency Virus (HIV), Hepatitis C Virus (HCV), Human Parainfluenza Virus Type 3 (HPIV3), Herpes Simplex Virus Type 1 (HSV1), Influenza A Virus (INFVA), Respiratory Syncytial Virus (RSV) and Severe Acute Respiratory Syndrome Coronavirus (SARS-CoV).

Considering the imbalance in the datasets, we incorporated the G-mean metric into our evaluation metrics. Table 6 presents the performance of our models on viral family classifications using an imbalance learning strategy. The model achieved promising results across six viral families, with accuracies ranging between 0.80 and 0.96, and G-mean scores between 0.86 and 0.93. Notably, the Paramyxoviridae family exhibited the best classification results with accuracy of 0.9625 and G-mean score of 0.9396. The same imbalance learning strategy is used for constructing models on targeted Virus classifications. Table 7 summarizes the performance on targeted viruses, which were classified effectively with accuracies ranging from 0.84 and 0.96 and G-mean scores ranging from 0.85 and 0.98. In particular, HPIV3 stands out with accuracy of 0.9641 and G-mean score of 0.9813. These results demonstrate our model’s effectiveness in accurately identifying AVPs targeting specific viral families or viruses.

**Table 6:** Performance summary on independent datasets of viral family datasets

Viral family	Accuracy	Sensitivity	Specificity	MCC	G-mean
Coronaviridae	0.8979	0.9348	0.8952	0.5709	0.9148
Flaviviridae	0.8378	0.9754	0.8070	0.6406	0.8872
Herpesviridae	0.8483	0.8806	0.8447	0.5199	0.8625
Orthomyxoviridae	0.8036	0.9655	0.7962	0.3654	0.8768
Paramyxoviridae	0.9625	0.9118	0.9682	0.8152	0.9396
Retroviridae	0.9009	0.9197	0.8897	0.7954	0.9046

**Table 7:** Performance summary on independent datasets of targeted virus datasets

Targeted virus	Accuracy	Sensitivity	Specificity	MCC	G-mean
HCV	0.8442	0.8818	0.8369	0.5913	0.8591
HIV	0.8818	0.8241	0.9089	0.7294	0.8654
HPIV3	0.9641	0.9999	0.9629	0.6786	0.9813
HSV1	0.8452	0.9630	0.8350	0.5161	0.8967
INFVA	0.8504	0.9643	0.8454	0.4131	0.9029
RSV	0.9414	0.9999	0.9386	0.6388	0.9688
SARS-CoV	0.8981	0.8857	0.8987	0.5005	0.8922
FIV	0.8559	0.9200	0.8534	0.3863	0.8861

#### Compare with other existing AVP prediction tools

To evaluate the performance of our proposed model, we compared with several existing models, including AVPpred[19], Chang’s method[20], AntiVPP 1.0[21], Meta-iAVP[22], DeepAVP[23], PreTP-Stack[51] and iAMPpred[52] on Set1 and Set2 datasets. The comparative results are summarized in Tables 8 and 9.

**Table 8:** Comparison of the performance of existing methods on on independent dataset of Set 1. Best performance values are in bold.

Method	Accuracy	Sensitivity	Specificity	MCC
AVPpred	0.8570	0.8830	0.8220	0.71
Chang et al.	0.8950	0.9170	0.8670	0.79
Meta-iAVP	0.8076	0.8333	0.7727	0.60
DeepAVP	0.8760	0.9000	0.8440	0.75
PreTP-Stack	0.8942	0.8666	0.9318	0.79
iAMPpred	0.8173	0.9000	0.7045	0.62
Our method	**0.9423**	**0.9333**	**0.9545**	**0.88**

**Table 9:** Comparison of the performance of existing methods on on independent dataset of Set 2. Best performance values are in bold.

Method	Accuracy	Sensitivity	Specificity	MCC
AVPpred	0.9250	0.9330	0.9170	0.85
Chang et al.	0.9330	0.9170	0.9500	0.87
AntiVPP 1.0	0.9300	0.8700	0.9700	0.87
Meta-iAVP	0.6949	0.8333	0.5517	0.40
DeepAVP	0.9330	**0.9670**	0.9000	0.87
PreTP-Stack	0.8475	0.8666	0.8276	0.70
iAMPpred	0.9152	0.9000	0.9310	0.83
Our method	**0.9576**	0.9333	**0.9828**	**0.92**

In Set 1, our model surpasses all comparative methods, achieving the highest accuracy of 0.9423, which is 4-14 percentage points higher than the other methods. Our method also leads significantly in terms of sensitivity and specificity, with values of 0.9333 and 0.9545, respectively. In terms of the MCC score, our model achieves 0.88, significantly higher than other models. Similarly, in Set 2, our model shows strong performance with an accuracy of 0.9576, sensitivity of 0.9333, specificity of 0.9828 and an MCC score of 0.92, outperforming other comparative methods. Although slightly inferior to DeepAVP in sensitivity, our model achieves a good balance between sensitivity and specificity, avoiding prediction bias.

Overall, our model performs excellently in identifying AVPs and non-AVPs, which can be attributed to several key factors: Firstly, we incorporated a comprehensive set of peptide features, including the composition, sequence order information, physicochemical properties, and evolutionary information of peptides, providing the model with multidimensional insights into peptides. Secondly, we employed contrastive learning technique, which helps the model to learn small but crucial differences by comparing same and different classes of peptide pairs, thus improving the model’s ability to differentiate between AVPs and non-AVPs. Moreover, by utilizing transformer, our model identifies relationships between features and reinforces important characteristics. Finally, through the adoption of a multi-feature fusion strategy, our model learns comprehensive features, which further improves its predictive accuracy and generalization performance.

### Performance comparison with other sequence encoding methods

To validate the superiority of our chosen sequence encodings, we evaluated our selected methods which include DistancePair, CKSAAGP, QSOrder and DDE against well-established methods including Amino Acid Composition (AAC), Di-Peptide Composition (DPC), Pseudo-Amino Acid Composition (PAAC), Composition of k-Spaced Amino Acid Pairs (CKSAAP), and the Composition, Transition, and Distribution of amino acids (CTDC, CTDT, CTDD). The comparison results are shown in Table S1, reveals that our chosen four feature extraction techniques outperform other common feature extraction methods in terms of overall performance. While DPC achieved the highest Sensitivity, our selected four encoding techniques demonstrated a more balanced performance with higher MCC and AUC values. Moreover, by integrating our selected four encodings, we further enhanced our model’s performance, proving that the combination of these features provides a better representation of peptides, thereby improving the model’s predictive capability.

### Performance comparison of individual models and feature fused model

**Figure 2:**
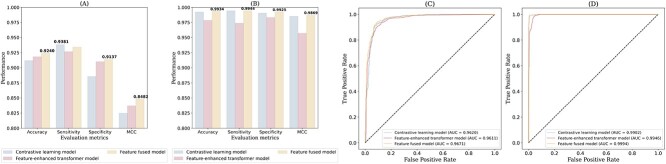
Performance comparison of individual models and feature fused model on (**A**,**C**) non-AVP dataset and (**B**,**D**) non-AMP dataset.

To demonstrate the effectiveness of our feature fusion strategy across different modules, we conducted a comparative analysis of models utilizing either the contrast learning module or the feature-enhanced transformer module alone with our feature fused model. As shown in Figure 2A and B, the feature fused model exhibited superior performance over the single-module models on both datasets. Specifically, on the non-AVP dataset, the feature fused model showed notable improvements in accuracy, specificity, and MCC score, reaching 0.9240, 0.9137 and 0.8482, respectively. Although slightly underperforming in sensitivity compared to the contrastive learning model, the feature fused model presented a more balanced and superior overall performance. On the non-AMP dataset, the feature fused model achieved the best performance in terms of accuracy, sensitivity, specificity, and MCC score, with values of 0.9934, 0.9944, 0.9925 and 0.9869, respectively. These outcomes not only highlight the efficiency of our method but also its robustness and adaptability across different types of data.

Moreover, as illustrated in Figure 2C and D, the receiver operating characteristic (ROC) curves for the two datasets show the yellow curve representing the feature fused model, achieving the highest area under the curve (AUC) in all comparisons. This result further validates the significant predictive superiority of the feature fused model.

In addition, in order to comprehensively evaluate the performance of the deep learning model in the classification task as well as its superiority in feature representation, we adopted the t-distributed stochastic neighbor embedding (t-SNE) technique for the visual presentation of positive and negative samples processed by the model in different modules[53]. The results show that the initial feature distribution is relatively disorganized and chaotic (Figure S3A and B). As the data passes through the model’s contrastive learning module as well as the feature-enhanced transformer module, the distinction between AVPs and non-AVPs becomes evident (Figure S3C and D). At the final prediction module, the separation between AVPs and non-AVPs was more obvious (Figure S3E), showcasing the effectiveness of our feature fusion strategy in enhancing classification accuracy.

### Ablation experiments

To investigate deeply the effect of the contrastive loss function in the contrastive learning module, the different encoding methods for peptide sequences, and the benefits of the transformer in the Feature-enhanced transformer module, we conducted the following three ablation studies:

(i) Comparison between contrast learning module with and without contrast loss function.
(ii) Comparison of different encoding methods in the input sequence of the Contrastive learning module: ordinal number encoding and Binary + BLOSUM62 + Zscale encodings.
(iii) Comparison between the Feature-enhanced transformer module with and without transformer.

**Figure 3:**
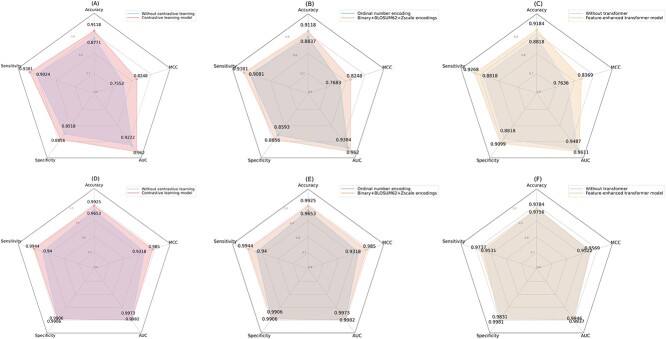
Performance comparison of ablation experiments on (**A-C**) non-AVP dataset and (**D-F**) non-AMP dataset.

As shown in Figure 3A and D, on both datasets, the model with the contrast loss function outperforms the model without it on all evaluation metrics. Notably, in the non-AVP dataset, scores for the five evaluation metrics were significantly higher for the contrastive loss model, showing a difference of 0.3–0.7 units compared to the control. The robust performance of contrast learning can be attributed to several factors: firstly, the contrastive loss function encourages the model to distinguish between positive and negative samples more explicitly, enhancing the model’s ability to capture crucial features. In addition, compared to conventional learning methods, the uniqueness of contrastive learning lies in its focus not only on learning the feature representation of individual samples but also on understanding the relative relationships between samples. This approach makes the model more stable and robust when dealing with diverse samples. Lastly, by emphasizing contrasts between samples, contrastive learning can effectively reduce the model’s excessive dependence on specific noise or local features, thereby enhancing its generalization capabilities.

As illustrated in Figure 3B and E, on both datasets, the concatenated encoding approach of binary, BLOSUM62 and Zscale evidently outperforms the method utilizing ordinal number encoding. This excellent performance can be attributed to the extensive information contained in the binary, BLOSUM62 and Zscale encoding methods. Specifically, the Binary profile offers a direct representation of the amino acid types and their sequences. The BLOSUM62 matrix reveals the likelihood of substitutions between different amino acids, enriching our understanding of the evolutionary trends in protein sequences. Meanwhile, the Zscale matrix captures in-depth the physical and chemical properties of amino acids, presenting the model with a more precise depiction of proteins.

As depicted in Figure 3C and F, upon the incorporation of the transformer, the model exhibits a notably enhanced performance on the non-AVP dataset. However, the improvement is relatively modest on the non-AMP dataset. This can be attributed to the fact that in the non-AMP dataset, positive and negative samples have already shown distinct differences after the initial encoding. For the non-AVP dataset, the introduction of the transformer evidently amplifies the representational power of features, allowing the model to better focus on key attributes, subsequently optimizing prediction outcomes.

### Model interpretation

The function of a protein is often related to its amino acids arrangement, and certain amino acids are particularly important due to their positions. By employing Deep Learning Important FeaTures (DeepLIFT)[54], a method for interpreting deep learning model predictions, we can analyze the positional importance of amino acids.

Considering that the majority AVPs exhibit sequence length distributions between 0 and 60, we divided these sequences into six groups: 0–10, 10–20, 20–30, 30–40, 40–50 and 50–60, to ensure a similar length distribution between positive and negative samples.

**Figure 4:**
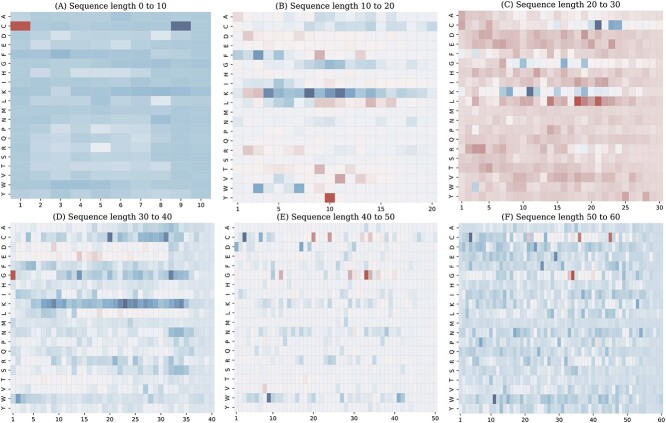
Amino acid position importance in different sequence length ranges. (**A**) Length range from 0 to 10. (**B**) Length range from 10 to 20. (**C**) Length range from 20 to 30. (**D**) Length range from 30 to 40. (**E**) Length range from 40 to 50. (**F**) Length range from 50 to 60.

In our study, we employed visualization techniques to show how the contrastive learning module processes various AVP sequences. In the heatmap, the more skewed blue color indicates more significant predictive impact on AVPs, and the more skewed red color indicates more important predictive impact on non-AVPs. As shown in Figure 4A, for sequences with lengths between 0–10, we observe that amino acid C contributes significantly to the AVP prediction at the ninth position, as well as to the non-AVP prediction at the first position. In Figure 4B–D, for sequences with lengths between 10–20, 20–30 and 30–40, respectively, we find that amino acid K contributes significantly to the prediction of the middle part of the sequence. In addition, amino acid Y at position 10 in the 10–20 range, amino acid L at positions 18 and 21 in the 20–30 range, and amino acid G at position 1 in the 30–40 range all have a significant effect on the prediction of non-AVPs. In Figure 4E, for sequences between 40–50 in length, we observe that C at position 3 and W at position 9 have a remarkable positive contribution to AVP prediction, whereas G at position 33 has a great impact on non-AVP prediction. Moreover, with the exception of C, D, G, F and W, the other amino acids have relatively small effects on most positions. Finally, in Figure 4F, for sequences between 50–60 in length, we note that C at position 4 and W at position 11 contribute considerably to AVP prediction, while C at positions 36 and 45 and G at position 34 have a significant contribution to non-AVP prediction.

Additionally, we employed Shapley Additive Explanations (SHAP) to explain the feature in our feature-enhanced transformer module [55]. Figure S4A and B summari zes e the contribution of each descriptor in the first stage of identification. The results show that the DDE descriptors exhibit considerable ability to represent antiviral activity, as evidenced by their significant overall impact and high average importance. This could be attributed to the significant difference in the frequency of certain dipeptides in AVPs compared to the expected values. Furthermore, CKSAAGP also shows high overall and average impacts, suggesting that common subsequences in AVPs may be critical regions for antiviral efficacy. Although features such as DistancePair and QSOreder have smaller dimensions and therefore contribute less to the overall impact, they still have considerable average significance. To investigate the critical features of AVPs, we selected the top 30 features from the entire feature set based on their importance and the results are shown in Figure S5. It can be observed that the DDE346 corresponds to the dipeptide ’VG’ (Valine and Glycine), the DDE355 to ’VR’ (Valine and Arginine), the DDE106 to ’GG’ (Glycine and Glycine), and the DDE106= to ’FS’ (Phenylalanine and Serine), with these DDE features contributing significantly to the prediction, indicating that the frequencies of these dipeptides show a significant deviation from their theoretical expected frequencies. Moreover, positively charged amino acids, particularly those paired with aliphatic spaces, are frequently among the significant features. This highlights the importance of positively charged amino acids and their interactions with aliphatic amino acids, which may be crucial in determining antiviral activity.

### Web interface

For user convenience, this framework has been implemented in a user-friendly interface(as shown in Figure S6), which is available for free at https://awi.cuhk.edu.cn/∼dbAMP/AVP/. Initially, users are required to input one or multiple protein sequences or upload a file containing sequences in fasta format. Subsequently, users can choose a model trained on different datasets. Following this, users can select the functional types of AVPs they wish to predict, specifically targeting different families or viruses. Afterward, users can click the ’Start prediction’ button to begin the prediction process. The prediction results are displayed in an intuitive format, showing the predicted AVPs and their potential functional types. Additionally, the datasets used in this study can be downloaded from the ’DOWNLOAD’ page.

## CONCLUSION

In recent years, AVPs have gained increasing attention as a potential method for viral therapy. However, identifying new AVPs through traditional wet-lab experiments is time-consuming, labor-intensive and costly. With the advancement of artificial intelligence, computational methods have accelerated the discovery of AVPs.

In that study, we introduced a two-stage predictor based on deep learning, which can identify AVPs and characterize their targets against different viruses at both the family and species levels. Our method has some significant advantages. Firstly, our proposed approach has the multi-functional types prediction of AVPs, which extends its application. Secondly, our model effectively captures subtle differences in peptide sequences through contrastive learning, enabling the model to separate different peptides by clustering similar peptides together. Additionally, by adopting a multi-feature fusion strategy, our model has learned comprehensive features, thereby enhancing the predictive accuracy of the model. Combined with DeepLIFT and SHAP, the predictive results of our model are more interpretable.

However, we also recognize its limitations. The effectiveness of our model heavily depends on the quality and diversity of the training dataset. Derived from existing databases, our dataset may not adequately encompass the full array of peptide sequences or functional types, potentially limiting performance on underrepresented viral families or targeted viruses. Moreover, due to sequence records falling below our established thresholds, our current model excludes certain viral families or targeted viruses due to insufficient positive sample sizes. As data availability improves in the future, we aim to expand our model to include these underrepresented groups, thereby enhancing its comprehensiveness and applicability. Additionally, the computational resource demands of our framework could pose limitations, especially in resource-limited settings, which might impact the model’s application. Lastly, while our imbalance learning strategy is effective, it may not completely address the issue of skewed sample distribution in certain categories. Exploring more methods to rectify this imbalance could lead to improvements in the model’s overall performance.

The experimental results demonstrated that our proposed framework surpassed current AVP prediction tools, achieving state-of-the-art performance. In addition, the interpretability of our model provided significant insights into peptide sequence features related to AVP recognition. This knowledge aided in the design of more targeted and effective AVPs to treat various viral diseases.

**Key Points**

- In this study, we proposed a two-stage computational framework for identifying antiviral peptides based on deep learning.
- Our model incorporates features from multiple perspectives, and the contrastive learning introduced effectively improves the representation of features.
- Our model not only can identifies AVPs from complex samples, but also recognizes AVPs targeting specific families or viruses.
- Comparative studies show that our method achieves state-of-the-art performance that outperforms existing AVP prediction tools.

## Supplementary Material

SUPPLEMENTARY_MATERIALS_bbae208

## Data Availability

Code and datasets of this study are available at https://github.com/GGCL7/AVP-IFT. Web server is available at https://awi.cuhk.edu.cn/∼dbAMP/AVP/.

## Author Biographies

**Jiahui Guan** is in the School of Medicine, The Chinese University of Hong Kong, Shenzhen, China, and also in the Kobilka Institute of Innovative Drug Discovery, The Chinese University of Hong Kong, Shenzhen, China.

**Lantian Yao** is in the Kobilka Institute of Innovative Drug Discovery, The Chinese University of Hong Kong, Shenzhen, China, and also in the School of Science and Engineering, The Chinese University of Hong Kong, Shenzhen, China.

**Peilin Xie** is in the Kobilka Institute of Innovative Drug Discovery, The Chinese University of Hong Kong, Shenzhen, China.

**Chia-Ru Chung** is in the Department of Computer Science and Information Engineering, National Central University, Taoyuan, Taiwan.

**Yixian Huang** is in the School of Medicine, The Chinese University of Hong Kong, Shenzhen, China.

**Ying-Chih Chiang** is currently an assistant professor in the Kobilka Institute of Innovative Drug Discovery, The Chinese University of Hong Kong, Shenzhen, China.

**Tzong-Yi Lee** is currently a professor in the Institute of Bioinformatics and Systems Biology, National Yang Ming Chiao Tung University, Hsinchu, Taiwan.
