# Supplementary material for: A two-stage computational framework for identifying antiviral peptides and their functional types based on contrastive learning and multi-feature fusion strategy
Source: Brief Bioinform. 2024 May 5;25(3):bbae208. doi: 10.1093/bib/bbae208 (PMC11070730; doi:10.1093/bib/bbae208)
Supplement: SUPPLEMENTARY_MATERIALS_bbae208 [file supplementary_materials_bbae208.pdf]

SUPPLEMENTARY MATERIALS

Supplementary Methods

Details of feature encoding algorithms:

1. Binary

The binary profile is a widely used technique for representing amino acid sequences. This method employs a one-hot encoding strategy to capture both the composition and order information of a given sequence. Specifically, each amino acid is represented as a 20-dimensional binary vector, such that only one dimension corresponds to the position of the encoded amino acid, while all other dimensions are set to zero.

2. BLOSUM62

In this dencoding, the BLOSUM62 matrix is employed to represent the protein primary sequence information as the basic feature set. A matrix comprising of m × n elements is used to represent each residue in a training dataset, where n denotes the peptide length and m = 20, which elements comprise 20 amino acids. Each row in the BLOSUM62 matrix is adopted to encode one of 20 amino acids. For instance, A is encoded as [4, −1, −2, −2, 0, −1, −1, −1, 0, −2, −1, −1, −1, −2, −1, 0, −3, −2, 0], R is encoded as [−1, 5, 0, −2, −3, 1, 0, −2, 0, −3, −2, 2, −1, −2, −1, −3, −2, −3], and so on.

3. Zscale

The Z-scale provides a quantitative representation of the physicochemical characteristics inherent to amino acids. Each residue is delineated by five metrics, capturing diverse physicochemical properties such as hydrophobicity and hydrophilicity, steric bulk properties and polarizability, polarity, and electronic influences. For instance, A is encoded as [0.24, −2.32, 0.60, −0.14, 1.30], C is encoded as [0.84, −1.67, 3.71, 0.18, −2.65], and so on.

4. DistancePair

The DistancePair encoding incorporates the amino acid distance pair coupling information and the amino acid reduced alphabet profile into the general pseudo amino acid composition vector. For the reduced alphabet profile, they are cp(13), cp(14), and cp(15) as defined below:

cp(13) = {MF; IL;V;A;C;WYQHP;G;T; S;N;RK;D; E}

cp(14) = {EIMV; L;F;WY;G; P;C;A; S; T;N;HRKQ; E;D}

sp(15) = {P;G; E;K;R;Q;D; S;N; T;H;C; I;V;W;YF;A; L;M}

where the single letters without a semicolon (;) to separate them mean belonging to a same cluster.

5. **CKSAAGP**

The Composition of *k*-Spaced Amino Acid Group Pairs (CKSAAGP) encoding is a variation of the CKSAAP encoding, which is our own proposal. It calculates the frequency of amino acid group pairs separated by any *k* residues (the default maximum value of *k* is set as 5). Taking *k* = 0 as an example, there are 25 0-spaced group pairs (i.e., *g1g1, g1g2, g1g3, ... g5g5*). Thus, a feature vector of CKSAAGP can be defined as:

$$\left(\frac{N_{g_1g_1}}{N_{\text{total}}}, \frac{N_{g_1g_2}}{N_{\text{total}}}, \frac{N_{g_1g_3}}{N_{\text{total}}}, \ldots, \frac{N_{g_5g_5}}{N_{\text{total}}}\right)_{25}$$

The value of each descriptor denotes the composition of the corresponding residue group pair in a protein or peptide sequence. For instance, if the residue group pair *g1g1* appears *m* times in the protein, the composition of the residue pair *g1g1* is equal to *m* divided by the total number of *0*- spaced residue pairs (N_{total}) in the protein. For *k* = 0, 1, 2, 3, 4 and 5, the values of N_{total} are $P-1$, $P-2$, $P-3$, $P-4$, $P-5$ and $P-6$ respectively, for a protein of length *P*.

6. QSOrder

For each amino acid type, a quasi-sequence-order descriptor can be defined as:

$$X_r = \frac{f_r}{\sum_{r=1}^{20} f_r + w\sum_{d=1}^{nlag} \tau_d}, \qquad r = 1, 2, \ldots, 20$$

where f_r is the normalized occurrence of amino acid type *r* and *w* is a weighting factor (*w* = 0.1), *nlag* and τ_d have the same definitions as described above. These are the first 20 quasi-sequence-order descriptors. The other 30 quasi-sequence-order descriptors are defined as:

$$X_d = \frac{w\tau_d - 20}{\sum_{r=1}^{20} f_r + w\sum_{d=1}^{nlag} \tau_d}, \qquad d = 21, 22, \ldots, 20 + nlag \cdot$$

7. DDE

The Dipeptide Deviation from Expected Mean (DDE) encoding offers a statistical perspective on the dipeptide distribution in proteins.It measures the deviation between the observed frequency of a dipeptide and its theoretically expected occurrence based on codon frequencies. DDE can reveal patterns or biases in protein composition. If a specific dipeptide appears more or less frequently than expected, it suggests that there might be evolutionary, functional, or structural reasons for this deviation. The DDE encoding is formulated by three parameters: dipeptide composition (D_c), theoretical mean (T_m), and theoretical variance (T_v). The above three parameters and the DDE are computed as follows. $D_c(r,s)$, the dipeptide composition measure for the dipeptide '*rs'*, is given as

$$Dc(r,s) = \frac{N_{rs}}{N}$$

where N_{rs} is the number of dipeptides represented by amino acid types r and s and N is the length of the protein or peptide. $T_m(r,s)$, the theoretical mean, is given

$$T_m(r,s) = \frac{C_r}{C_N} \times \frac{C_s}{C_N}$$

where C_r is the number of codons that code for the first amino acid and C_s is the number of codons that code for the second amino acid in the given dipeptide '*rs*'. C_N is the total number of possible codons, excluding the three stop codons (i.e., 61). T_v (r,s), the theoretical variance of the dipeptide '*rs*', is given by:

$$T_v(r,s) = \frac{T_m(r,s)(1 - T_m(r,s))}{N-1}$$

Finally, *DDE(r,s)* is calculated as:

$$DDE(r,s) = \frac{Dc(r,s) - Tm(r,s)}{\sqrt{Tv(r,s)}}$$

Supplementary Figures

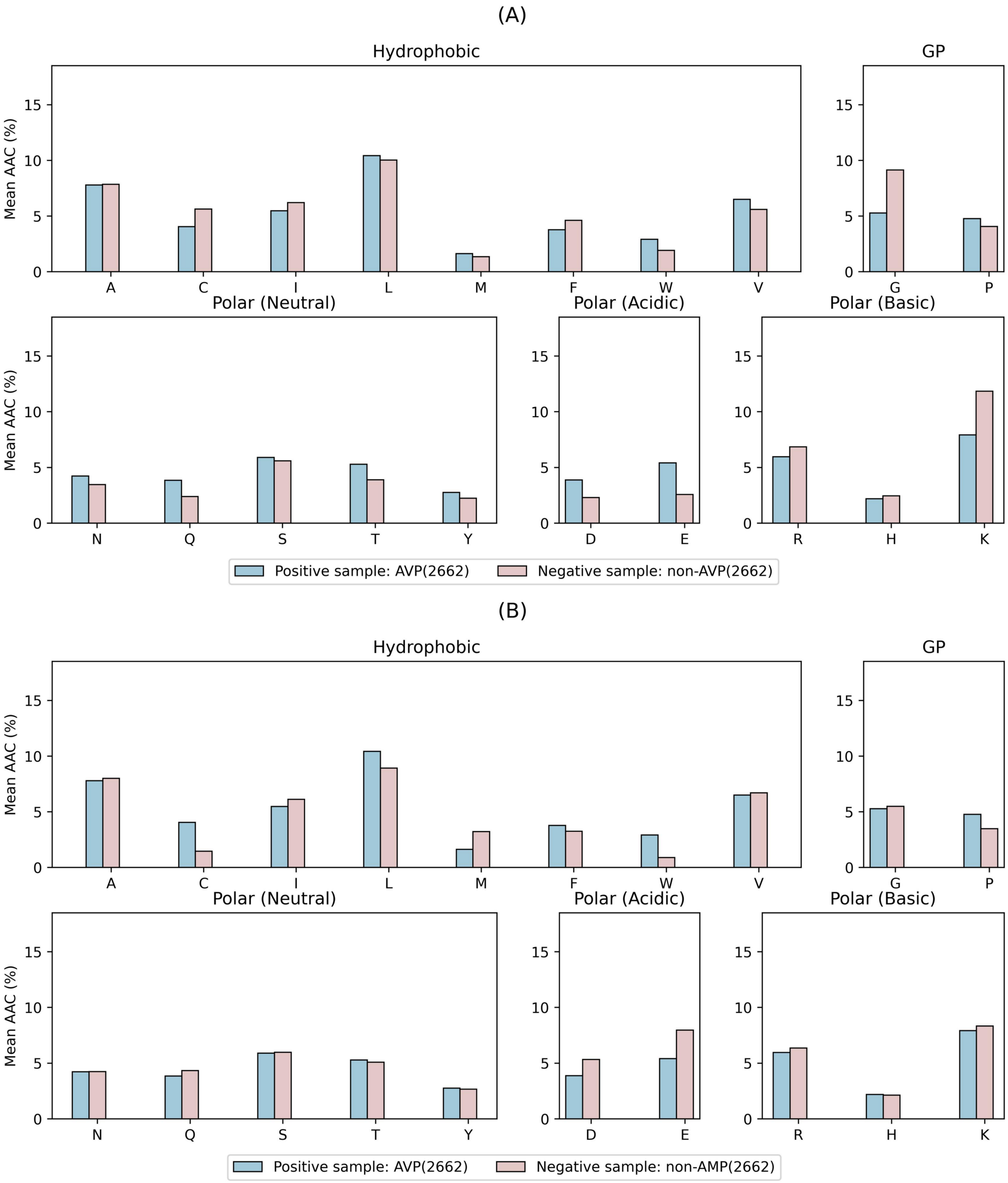

Figure S1. Mean amino acid composition of positive and negative samples on (A) non-AVP and (B) non-AMP datasets.

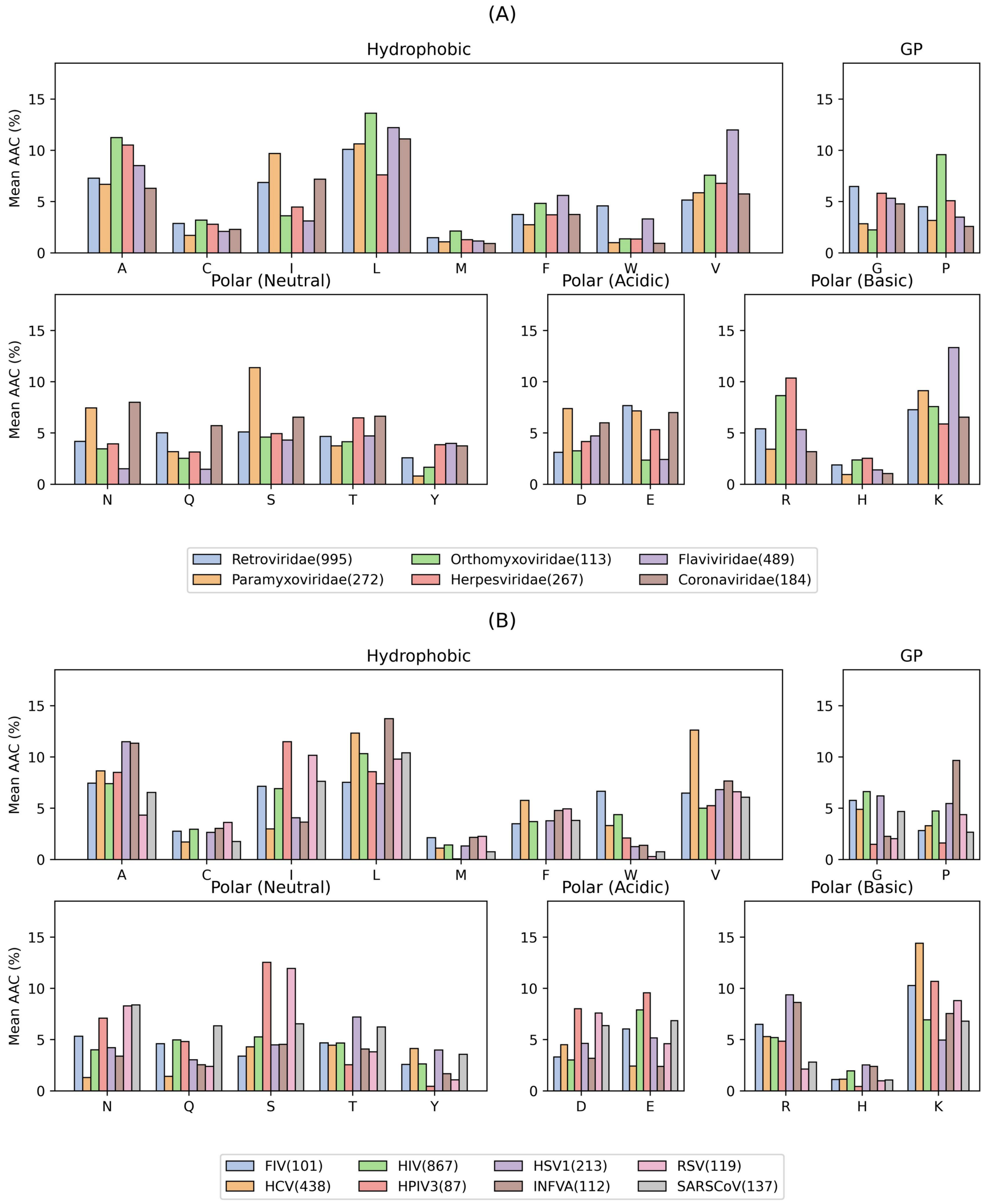

Figure S2. Mean amino acid composition of AVPs against (A) six key viral families (Coronaviridae, Retroviridae, Herpesviridae, Paramyxoviridae, Orthomyxoviridae, Flaviviridae) and (B) eight viruses (FIV, HCV, HIV, HPIV3, HSV1, INFVA, RSV, SARS-CoV).

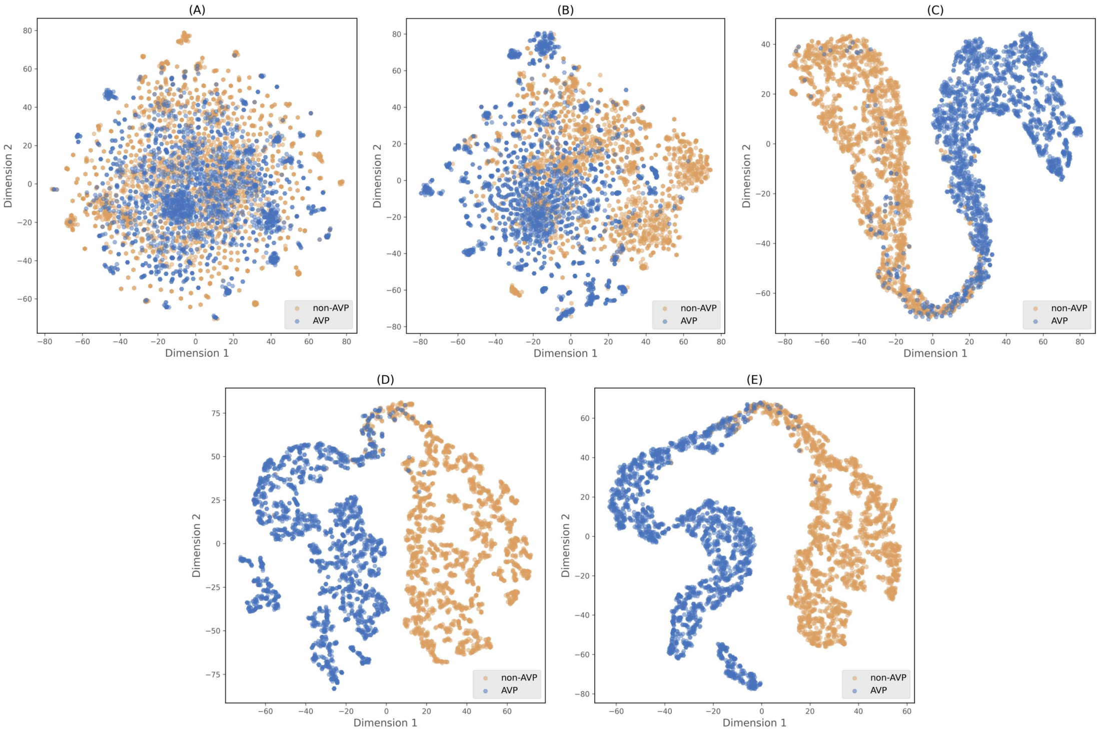

Figure S3. Visualization of positive and negative samples in different modules of the model on non-AVP dataset. The visualized modules include (A) and (B) Input features (C) Contrastive learning module (D) Feature-enhanced transformer module (E) Prediction module. The blue dots denote AVPs and the yellow dots denote non-AVPs.

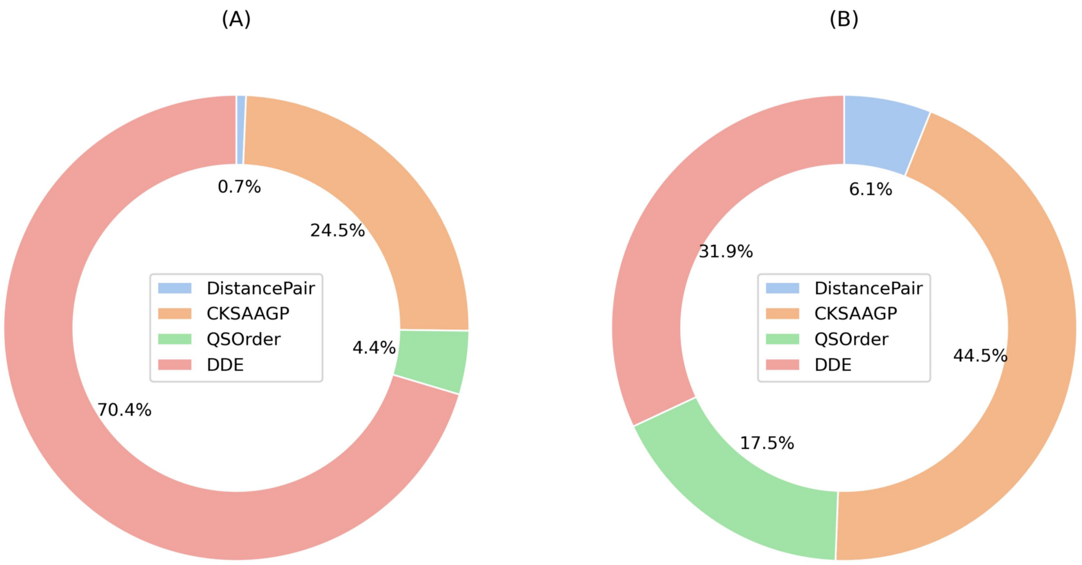

Figure S4. Contribution of different encodings in the feature-enhanced transformer module to first-stage identification. (A) Ratio of the total impact of different peptide encodings on the prediction. (B) The normalized average feature importance associated with the dimension of that peptide encoding.

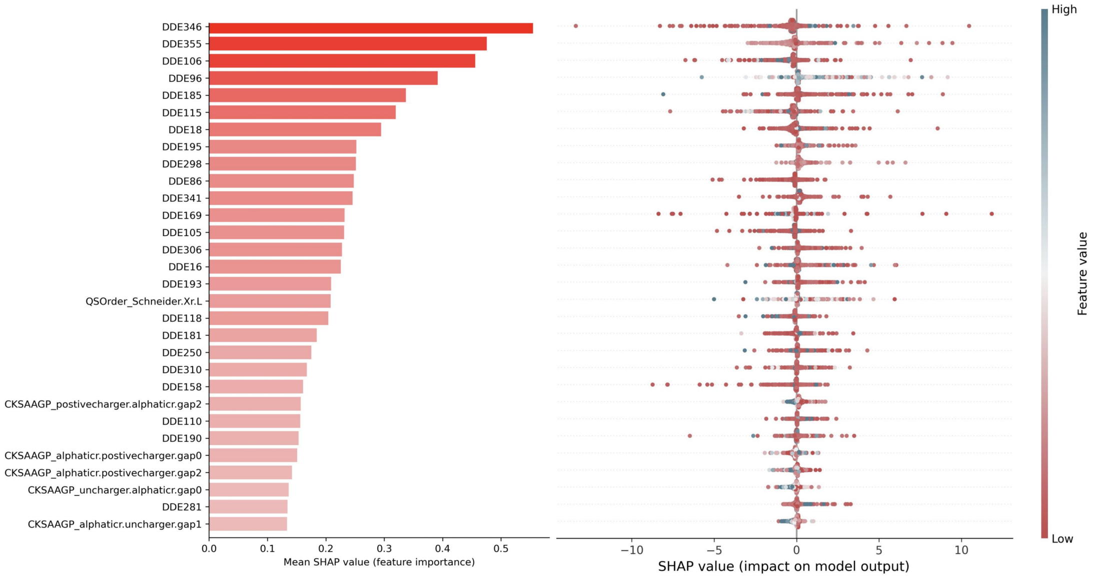

Figure S5. The top 30 important features in the first stage of identification of the feature-enhanced transformer module. The left bar plot represents the feature importance calculated by the averaged absolute Shapley value. The right beeswarm plot gives the effect of different feature values on the prediction.

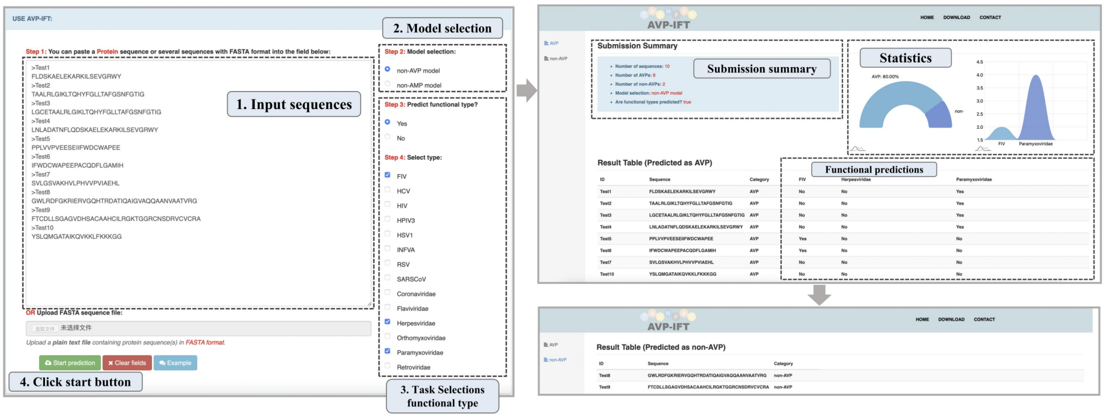

Figure S6. Diagram of the web interface using example sequences. This includes the following steps: sequences input, model selection, function type selection and prediction. The prediction results page will then be displayed, including the submission summary, results statistics and results display.

Supplementary Tables

Table S1. Performance comparison between our sequence encodings and some common feature extraction methods on non-AVP dataset.

Feature	Accuracy	Sensitivity	Specificity	MCC	AUC
AAC	0.8105	0.7786	0.8424	0.6223	0.8897
DPC	0.8462	0.9531	0.7392	0.7087	0.9295
PAAC	0.8227	0.9193	0.7260	0.6578	0.8884
CKSAAP	0.8340	0.8799	0.7879	0.6708	0.6416
CTDC	0.7974	0.7411	0.8537	0.5986	0.8781
CTDT	0.8124	0.7730	0.8518	0.6267	0.9073
CTDD	0.8077	0.9250	0.6904	0.6330	0.8738
DistancePair	0.8527	0.8499	0.8555	0.7054	0.9074
CKSAAGP	0.9081	0.9043	0.9118	0.8161	0.9620
QSOrder	0.8630	0.8724	0.8536	0.7262	0.9188
DDE	0.8668	0.8593	0.8743	0.7337	0.9309
DistancePair+CKSAAGP+QSOrder+DDE	0.9184	0.9268	0.9099	0.8369	0.9611

Table S2. Performance summary on independent dataset of non-AVP-unbalanced and non-AMP-unbalanced datasets.

Dataset	Accuracy	Sensitivity	Specificity	MCC	AUC
non-AVP-unbalanced	0.9171	0.8630	0.9453	0.8148	0.9691
non-AMP-unbalanced	0.9882	0.9887	0.9880	0.9742	0.9990

In this study, we also trained models without reducing the number of negative samples, which total 5116 for the non-AVP-unbalanced dataset and 4979 for the non-AMP-unbalanced dataset.

Table S3. Performance comparison of individual models and feature fused model on non-AVP dataset.

Model	Accuracy	Sensitivity	Specificity	MCC	AUC
Contrastive learning model	0.9118	0.9381	0.8856	0.8248	0.9620
Feature-enhanced transformer model	0.9184	0.9268	0.9099	0.8369	0.9611
Feature fused model	0.924	0.9343	0.9137	0.8482	0.9671

Table S4. Performance comparison of individual models and feature fused model on non-AMP dataset.

Model	Accuracy	Sensitivity	Specificity	MCC	AUC
Contrastive learning model	0.9925	0.9944	0.9906	0.985	0.9982
Feature-enhanced transformer model	0.9784	0.9737	0.9831	0.9569	0.9946
Feature fused model	0.9934	0.9944	0.9925	0.9869	0.9994

Table S5. Performance comparison between contrast learning module with and without contrast loss function on non-AVP dataset and non-AMP dataset.

Model	Dataset	Accuracy	Sensitivity	Specificity	MCC	AUC
without contrast loss function	non-AVP	0.8771	0.9024	0.8518	0.7552	0.9222

with contrast loss function	non-AVP	0.9118	0.9381	0.8856	0.8248	0.9620
without contrast loss function	non-AMP	0.9400	0.8874	0.9925	0.8848	0.9979
with contrast loss function	non-AMP	0.9925	0.9944	0.9906	0.9850	0.9982

Table S6. Performance comparison of different encoding methods in the input sequence of the Contrastive learning module: ordinal number encoding and Binary + BLOSUM62 + Zscale encodings on non-AVP dataset and non-AMP dataset.

Encoding	Dataset	Accuracy	Sensitivity	Specificity	MCC	AUC
ordinal number encoding	non-AVP	0.8837	0.9081	0.8593	0.7683	0.9384
Binary + BLOSUM62 + Zscale encodings	non-AVP	0.9118	0.9381	0.8856	0.8248	0.9620
ordinal number encoding	non-AMP	0.9653	0.9400	0.9906	0.9318	0.9973
Binary + BLOSUM62 + Zscale encodings	non-AMP	0.9925	0.9944	0.9906	0.9850	0.9982

Table S7. Performance comparison between the Feature-enhanced transformer module with and without transformer on non-AVP dataset and non-AMP dataset.

Model	Dataset	Accuracy	Sensitivity	Specificity	MCC	AUC
without transformer	non-AVP	0.8818	0.8818	0.8818	0.7636	0.9487
with transformer	non-AVP	0.9184	0.9268	0.9099	0.8369	0.9611
without transformer	non-AMP	0.9756	0.9531	0.9981	0.9522	0.9937
with transformer	non-AMP	0.9784	0.9737	0.9831	0.9569	0.9946
